# Integrating systemic inflammation and liver biomarkers: prognostic implications of the ferritin index in heart failure

**DOI:** 10.1080/07853890.2025.2540020

**Published:** 2025-08-01

**Authors:** Ching-Hui Huang, Chew-Teng Kor, Chia-Chu Chang

**Affiliations:** a Division of Cardiology, Department of Internal Medicine, Changhua Christian Hospital, Changhua, Taiwan; b Department of Mathematics, National Changhua University of Education, Changhua, Taiwan; c Department of Beauty Science and Graduate Institute of Beauty Science Technology, Chienkuo Technology University, Changhua, Taiwan; d Graduate Institute of Clinical Medicine, College of Medicine, National Chung Hsing University, Taichung, Taiwan; e Big Data Center, Changhua Christian Hospital, Changhua, Taiwan; f Graduate Institute of Statistics and Information Science, National Changhua University of Education, Changhua, Taiwan; g Department of Internal Medicine, Kuang Tien General Hospital, Taichung, Taiwan; h Department of Nutrition, Hungkuang University, Taichung, Taiwan

**Keywords:** Heart failure, ferritin index, fibrosis-4 (FIB-4) score, major adverse cardiovascular events, metabolic hyperferritinaemia

## Abstract

**Background:**

Heart failure (HF) is increasingly recognized as a multisystem syndrome involving systemic inflammation and metabolic dysfunction in addition to cardiac and hepatic impairment. Traditional liver fibrosis scores, such as the fibrosis-4 (FIB-4) index, focus solely on hepatic injury and may underestimate the broader systemic burden. The ferritin index, which adjusts serum ferritin for age and sex, may better reflect inflammatory and metabolic dysregulation, including features of metabolic hyperferritinaemia. This study aimed to evaluate the prognostic value of the ferritin index versus the FIB-4 score in predicting major adverse cardiovascular events (MACE) in patients with HF.

**Materials and methods:**

This retrospective cohort study included 751 HF patients from the Changhua Christian Hospital Clinical Research Database, spanning all ejection fraction categories. Cox regression models were used to assess associations between MACE and tertiles of ferritin index and FIB-4 score, with adjustments for baseline characteristics.

**Results:**

Patients in the highest ferritin index tertile had significantly increased MACE risk (adjusted hazard ratio 1.92; *p* = 0.003). This association remained robust in the sensitivity and subgroup analyses. The FIB-4 score was not significantly associated with MACE. Subgroup analysis showed stronger associations in patients aged ≥70 years, with higher BMI (≥24 kg/m^2^), reduced EF, low FIB-4 (<1.45), and abnormal hemoglobin or iron levels.

**Conclusion:**

The ferritin index outperforms the FIB-4 score in predicting MACE in HF patients. By integrating systemic inflammatory and metabolic signals, it improves risk stratification, especially in those with features of metabolic hyperferritinaemia.

## Introduction

Emerging evidence highlights the need to move beyond traditional disease silos and view cardiovascular, kidney, liver, and metabolic health as an interconnected continuum [1,2]. Heart failure (HF), once conceptualized primarily as a cardiac disorder, is now acknowledged as a systemic syndrome influenced by complex interactions among multiple organ systems. This is particularly true for HF with preserved ejection fraction (HFpEF), whose prevalence is increasing, surpassing that of HF with reduced ejection fraction (HFrEF) [3]. Within this multisystem context, hepatic dysfunction has gained increasing recognition as both a consequence and a contributor to HF pathophysiology. Right-sided or biventricular HF commonly results in hepatic congestion and hypoperfusion, leading to a spectrum of liver abnormalities ranging from mild biochemical disturbances to advanced fibrosis [4,5]. Liver involvement is especially pronounced in advanced HF stages, where it may accelerate disease progression [5,6]. Several non-invasive scoring systems, including the fibrosis-4 (FIB-4) index—originally developed to assess liver fibrosis in viral hepatitis—have been applied in HF populations to quantify hepatic involvement. Although FIB-4 has demonstrated associations with mortality and cardiovascular events in HFpEF and acute HF [7–9], it remains a liver-specific marker and may not adequately capture the broader systemic inflammatory and metabolic alterations observed in HF.

Systemic inflammation is a key feature of both acute and chronic HF, with increased neurohormonal and inflammatory activation [10]. Serum ferritin, an iron-storage protein and acute-phase protein, rises in response to inflammation and oxidative stress. The EDIFICA cohort demonstrated that elevated ferritin levels independently predict adverse outcomes in acute HF [11], irrespective of traditional HF prognostic markers. Notably, hyperferritinaemia, even in the absence of iron overload, is frequently observed in metabolic dysfunction-associated steatotic liver disease (MASLD) [12] and correlates with both hepatic and cardiometabolic disease severity [13,14].

Recent consensus has introduced the concept of metabolic hyperferritinaemia (MHF), defined as elevated serum ferritin in the context of metabolic dysfunction, such as obesity, type 2 diabetes, or dyslipidemia, without classical iron overload [15]. MHF reflects a pathophysiological state of inflammation-driven ferritin elevation, preserved hepcidin levels, and iron sequestration in macrophages—hallmarks of metabolic dysregulation [16,17]. It is increasingly recognized as a distinct phenotype with implications for both hepatic and cardiovascular risk stratification.

This trio of triads: hepatic dysfunction, systemic inflammation, and iron sequestration is increasingly recognized as a unified axis in HF, where inflammatory signaling promotes hepcidin-mediated iron retention in macrophages, disrupts hepatic iron export, and contributes to myocardial remodeling, fibrosis, and metabolic stress [18–23]. This mechanistic interplay provides a biologically plausible basis for using integrative biomarkers to assess HF risk beyond traditional cardiac parameters.

The ferritin index, which normalizes serum ferritin values to age- and sex-specific reference limits, was developed to enhance the interpretability of ferritin levels and to better capture the underlying systemic milieu. Unlike FIB-4, the ferritin index incorporates inflammatory and metabolic signals alongside hepatic involvement, potentially offering a more comprehensive marker of multisystem burden in HF. Comparing these two indices is essential to identify which biomarker more accurately predicts outcomes in HF, particularly in patients with overlapping metabolic and inflammatory comorbidities that may be underrepresented by liver-specific tools.

Accordingly, this study aims to compare the prognostic value of the ferritin index versus the FIB-4 score for predicting major adverse cardiovascular events (MACE) in patients with HF across different ejection fraction (EF) categories. We hypothesize that the ferritin index, by integrating systemic inflammation, metabolic dysfunction, and hepatic biomarkers, offers superior risk stratification compared with the FIB-4 score.

## Methods

### Data source

This retrospective observational study was conducted at Changhua Christian Hospital (CCH), a medical center in Taiwan. Data were retrieved from the Changhua Christian Hospital Clinical Research Database (CCHRD), with all personal identifiers encrypted before analysis. The study was approved by the Institutional Review Board of CCH (approval number 230422) and was exempt from full review, with informed consent waived. The study included patients admitted to the hospital or followed in outpatient clinics between January 2011 and December 2022.

### Study population

A total of 3,293 patients with heart failure (HF) were initially selected. HF was diagnosed by a cardiologist based on ICD-9-CM (428) or ICD-10-CM (I50) codes, with NT-proBNP ≥125 pg/ml during hospitalization or outpatient follow-up and a complete echocardiographic evaluation recorded in CCHRD. The index date was defined as the first recorded HF diagnosis.

Ferritin testing was part of routine laboratory assessments, and patients with valid ferritin results were included. A total of 2,542 patients were excluded due to missing ferritin data, leaving 751 eligible patients for analysis. Serum ferritin was measured using the Beckman two-site immunoenzymatic assay, with results adjusted for age and sex based on Beckman Coulter’s reference intervals [24]. The ferritin index (FI) was calculated as: FI = observed ferritin level/upper limit of normal level for age and sex [25]. The fibrosis-4 (FIB-4) score, a non-invasive marker of liver stiffness, was calculated using: age (years) × AST (U/L)/(platelet count (10^9^/L) × ALT (U/L)^1/2^).

FIB-4 categories were defined as **<**1.45 (minimal stiffness), 1.45–3.25 (moderate stiffness), and >3.25 (advanced stiffness) [26,27]. Due to the lack of established ferritin index cutoffs in heart failure populations, tertiles were used to pragmatically stratify patients while ensuring adequate subgroup sizes. This exploratory approach reflects the novelty of the ferritin index in this context and follows similar methodology in early biomarker studies.

**Figure 1.**
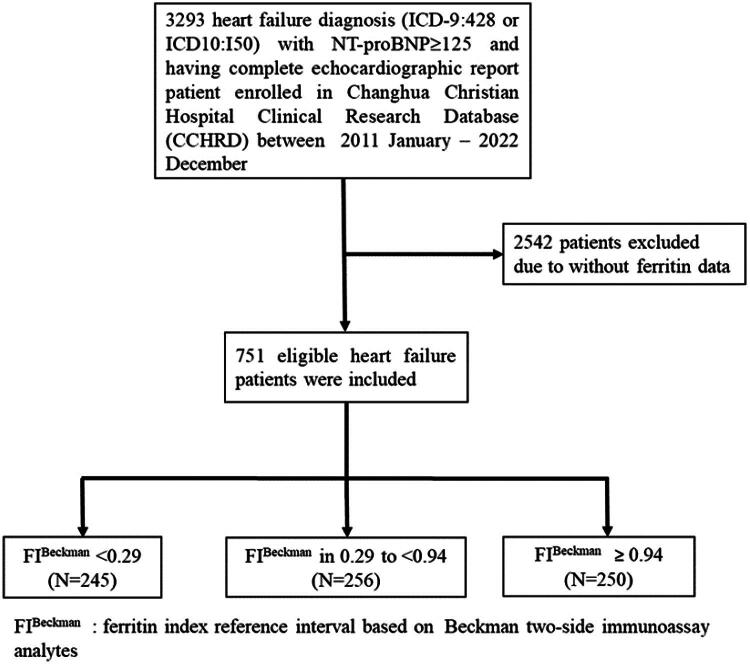
Study flow chart.

A flowchart (Figure 1) illustrates the cohort selection process.

### Outcome measures and variables

The primary endpoint was MACE, defined as a composite of:

- HF hospitalization
- Acute myocardial infarction
- Stroke (ischemic or hemorrhagic)
- New-onset atrial fibrillation (AF)

Patients were followed from the index date until the first occurrence of MACE or December 2022. Comorbidities, including hypertension, diabetes, chronic kidney disease (CKD), hyperlipidemia, chronic obstructive pulmonary disease (COPD), and AF, were identified *via* ICD-9-CM/ICD-10-CM codes. Medication use (antidiabetics, statins, antihypertensives, calcium channel blockers, beta-blockers, and diuretics) was recorded based on World Health Organization Anatomical Therapeutic Chemical (ATC) codes, with users defined as those on medication for ≥28 consecutive days before MACE occurrence.

### Statistical analysis

Continuous variables were expressed as mean ± standard deviation, and categorical variables as counts (percentages). One-way ANOVA, Chi-square, or Fisher’s exact tests were used for statistical comparisons.

To minimize confounding, an inverse probability weighting (IPW) model with propensity scores was applied, balancing baseline characteristics across ferritin index tertiles. To address potential confounding, we employed inverse probability of treatment weighting (IPTW) using propensity scores derived from a multinomial logistic regression model. The exposure variable was the ferritin index, categorized into tertiles (high, medium, and low). All baseline covariates listed in Table 1 were included as predictors in the propensity score model. IPTW weights were calculated as the inverse of the probability of each individual’s observed ferritin group assignment. Specifically:

- For individuals in the high ferritin group: weight = 1/(propensity score for high ferritin index)
- For those in the medium group: weight = 1/(propensity score for medium ferritin index)
- For the low group: weight = 1/(propensity score for low ferritin index)

**Table 1.** Demographic and clinical characteristics of the study cohort, categorized by ferritin index tertiles.

	FI^Beckman^<0.29	FI^Beckman^ in 0.29 to <0.94	FI^Beckman^ ≥ 0.94	*p* value before IPTW	*p* value after IPTW
Sample size	245	256	250		
LVEF(%)	47.9 ± 17.5	50.1 ± 17.1	50.4 ± 16.4	0.209	0.947
Heart failure type					
HFrEF	94(38.37%)	85(33.2%)	77(30.8%)	0.129	0.111
HFmrEF	34(13.88%)	27(10.55%)	41(16.4%)		
HFpEF	117(47.76%)	144(56.25%)	132(52.8%)		
Age(years)	72.7 ± 16.4	73.2 ± 13.3	71.7 ± 13.4	0.531	0.528
Gender, male	99(40.41%)	125(48.83%)	137(54.8%)	0.006	0.740
BMI (kg/m^2^)	25.5 ± 12.3	25.3 ± 5.1	24.5 ± 4.9	0.340	0.297
NT_proBNP(pg/mL)	8441 ± 8700.1	9435.5 ± 8348.2	10236.8 ± 8695.9	0.067	0.804
FIB-4 score	7.3 ± 14.4	6.9 ± 10.8	9.3 ± 26.3	0.307	0.380
<1.45	60(24.5%)	51(19.9%)	38(15.2%)	0.067	0.074
1.45 − 3.25	58(23.67%)	65(25.39%)	85(34%)		
>3.25	127(51.84%)	140(54.69%)	127(50.8%)		
Comorbidity disease					
DM	21(8.57%)	15(5.86%)	20(8%)	0.474	0.664
Hypertension	155(63.27%)	175(68.36%)	172(68.8%)	0.348	0.779
Hyperlipidemia	95(38.78%)	116(45.31%)	83(33.2%)	0.020	0.831
Coronary artery disease	86(35.1%)	78(30.47%)	82(32.8%)	0.543	0.538
COPD	42(17.14%)	44(17.19%)	41(16.4%)	0.966	0.964
CKD	93(37.96%)	120(46.88%)	133(53.2%)	0.003	0.920
Atrial fibrillation	68(27.76%)	67(26.17%)	62(24.8%)	0.756	0.836
Stroke	50(20.41%)	58(22.66%)	51(20.4%)	0.774	0.626
Medication use in hypertension					
ACEARB	205(83.67%)	213(83.2%)	210(84%)	0.971	0.683
Alpha blocker	24(9.8%)	37(14.45%)	31(12.4%)	0.282	0.942
Beta blocker	172(70.2%)	196(76.56%)	188(75.2%)	0.235	0.828
Calcium channel blocker	143(58.37%)	184(71.88%)	158(63.2%)	0.006	0.745
Thiazide	31(12.65%)	38(14.84%)	33(13.2%)	0.756	0.989
Loop diuretics	219(89.39%)	237(92.58%)	205(82%)	0.001	0.893
Spironolactone	117(47.76%)	116(45.31%)	91(36.4%)	0.027	0.702
Anti-DM medication	119(48.57%)	134(52.34%)	127(50.8%)	0.128	0.746
Statin	105(42.86%)	100(39.06%)	91(36.4%)	0.336	0.989
Aspirin	180(73.47%)	193(75.39%)	215(86%)	0.001	0.731
NSAID	7(2.86%)	9(3.52%)	10(4%)	0.784	0.626
Lab data					
HbA1c (%)	6.7 ± 1.5	6.7 ± 1.2	6.6 ± 1.4	0.597	0.573
Hb (g/dL)	9.6 ± 2.2	9.9 ± 2.2	9.3 ± 3.4	0.038	0.545
Creatinine (mg/ dL)	2.6 ± 2.7	3.4 ± 3.5	4.4 ± 3.7	<0.001	0.051
Albumin (g/dL)	3.1 ± 0.5	3.1 ± 0.5	3 ± 0.9	0.562	0.751
WBC count (10^3^ /μL)	8.7 ± 3.9	9.5 ± 4.7	10 ± 7.6	0.031	0.791
K (mmol/L)	4.2 ± 0.8	4.2 ± 0.8	5 ± 8.7	0.130	0.404
Estimated GFR (mL/min/1.73 m^2^)	44.9 ± 34.8	36.2 ± 31.6	34.5 ± 39.9	0.002	0.575
Na (mmol/L)	135.3 ± 7.1	134.7 ± 6	133.9 ± 11.6	0.161	0.920
Platelet count (10^3^ /μL)	229.2 ± 99.8	210.9 ± 98.1	195.4 ± 110	0.001	0.645
RDW (%)	17 ± 3.3	15.9 ± 2.9	16.6 ± 4.1	0.001	0.327
GPT(ALT) (U/L)	27.2 ± 39.7	29.2 ± 52.3	56.6 ± 237.5	0.036	0.166
GOT(AST) (U/L)	47.3 ± 105.2	37.4 ± 104.2	80.3 ± 325.1	0.051	0.541
Cholesterol (mg/dL)	152.2 ± 33.9	159.3 ± 41.3	153.4 ± 48.2	0.119	0.891
Triglyceride (mg/dL)	122.2 ± 89.4	130 ± 81.7	121.5 ± 67.2	0.409	0.701
LDL cholesterol (mg/dL)	86 ± 24.9	90.7 ± 32.5	86.8 ± 37.1	0.209	0.913
HDL cholesterol (mg/dL)	39.9 ± 9.8	40.5 ± 9.8	38.6 ± 12	0.113	0.934
APTT (sec.)	32.8 ± 7.4	33.3 ± 9	32.8 ± 12	0.815	0.538
Ferritin (ng/ml)	38.2 ± 24.2	184.2 ± 60.9	750 ± 700	<0.001	<0.001
Fe (μg/dL)	40.7 ± 30.6	51 ± 27.8	57.6 ± 36.4	<0.001	<0.001
Neutrophil-to-lymphocyte ratio (NLR)	7.58 ± 10.78	9.86 ± 13.9	9.77 ± 13.38	0.080	0.612
Lymphocyte-to-monocyte ratio (LMR)	2.61 ± 2.59	2.12 ± 1.68	2.52 ± 3.69	0.114	0.804

FI^Beckman^: ferritin index reference interval based on Beckman two-side immunoassay analytes.

These stabilized weights were then applied in Cox proportional hazards models to estimate the association between ferritin index and MACE outcomes while accounting for measured confounders. For subgroup analyses involving combined ferritin and FIB-4 categories, we used the same IPTW approach. A multinomial model incorporating all baseline covariates was used to estimate the joint probability of ferritin/FIB-4 group assignment, and the resulting weights were applied in the outcome models accordingly. Restricted cubic splines assessed the relationship between ferritin index, FIB-4, and MACE as continuous variables. Kaplan–Meier analysis compared cumulative MACE incidence across groups. Given the competing risk of death, IPW-adjusted Cox models incorporating the Fine and Gray method were used to estimate hazard ratios (HRs) with 95% confidence intervals (CIs), adjusting for demographics, comorbidities, medications, and laboratory data.

### Sensitivity and subgroup analyses

Sensitivity analyses excluded patients with pre-existing coronary artery disease, stroke, atrial fibrillation, or arrhythmias. Subgroup analyses examined interactions with key factors, including ejection fraction type, NT-proBNP, hemoglobin levels (normal: women 11–16 g/dL, men 13–18 g/dL), iron levels (50–212 µg/dL), FIB-4 scores, and age ≥75 years. A forest plot illustrated interaction effects.

All statistical analyses were performed using SAS version 9.4, with visualization implemented in R version 4.2.0 (ggplot2 and forest package). A two-tailed *p* < 0.05 was considered statistically significant.

## Results

### Patient characteristics

The final study cohort included 751 patients, with a mean age of 72.5 ± 14.4 years, and 361 (48%) were male. There were 539 outpatients (71.77%) and 212 inpatients (28.23%). Based on the ferritin index tertiles, patient characteristics were compared (Table 1). Heart failure with preserved LVEF was present in 393 patients (52.3%), while 256 patients (34.1%) had heart failure with reduced LVEF. The remaining 102 patients (13.6%) had HF with mildly reduced LVEF. Patients in the highest ferritin index tertile (≥0.94) had numerically higher NT-proBNP levels than those in the lower tertiles, though the differences did not reach statistical significance. The FIB-4 score and comorbidities were similar across groups. A total of 394 subjects (53.5%) had a FIB-4 score >3.25. Medications, laboratory data, and inflammatory parameters were comparable among the three groups after IPTW adjustment, except for iron metabolism parameters. Notably, patients in the lowest ferritin index tertile (<0.29) had significantly lower serum iron and ferritin levels compared to other groups, even after IPTW adjustment. For transparency, Supplementary Table 1 presents a comparison of demographic and clinical characteristics between the included cohort (*n* = 751) and the excluded cohort (*n* = 2,542).

### Ferritin, ferritin index, and major adverse cardiovascular events (MACE)

**Figure 2.**
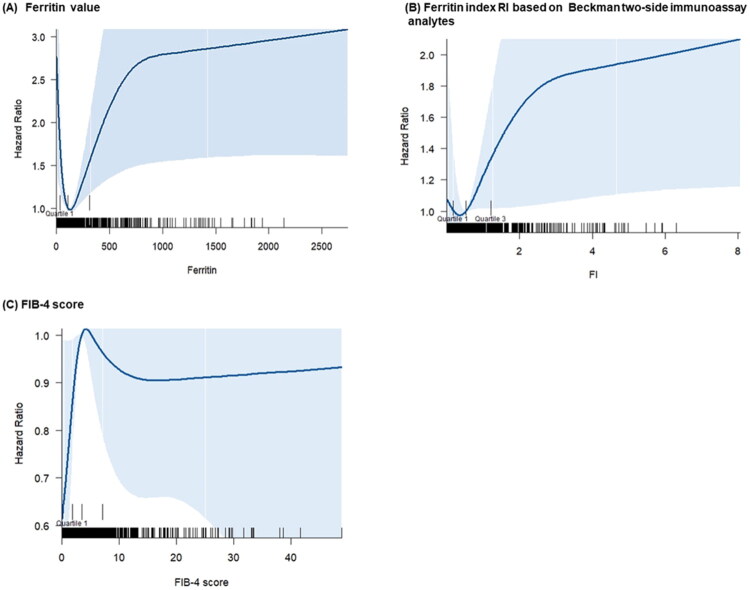
Restricted cubic curves illustrating the association between ferritin markers, FIB-4 scores, and MACE risk.

**Figure 3.**
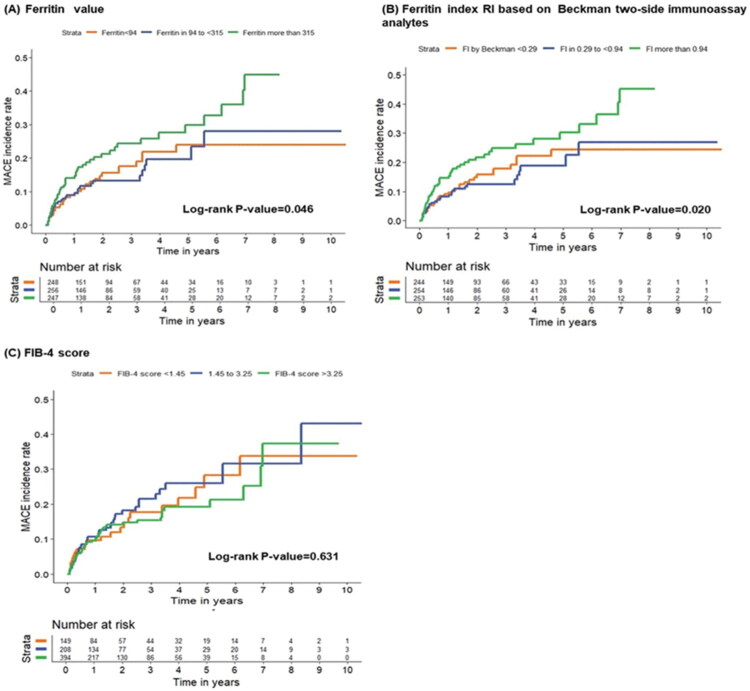
Kaplan–Meier curves illustrating MACE incidence rates among ferritin markers and FIB-4 score tertiles.

Analysis using restricted cubic splines did not show a linear correlation between ferritin levels, ferritin index, and MACE in heart failure patients (Figure 2A,B). Kaplan–Meier survival analysis demonstrated that patients in the highest ferritin tertile and ferritin index groups had an increased risk of MACE, with a median follow-up of 1.48 years (IQR: 0.57–3.44 years) (log-rank test, *p* = 0.046 and *p* = 0.020, respectively) (Figure 3A,B).

Table 2 outlines the impact of serum ferritin and ferritin index on MACE rates. The highest ferritin tertile was significantly associated with elevated MACE event rates (adjusted HR: 1.61, *p* = 0.038), with even stronger associations after IPTW adjustment (HR: 1.67, *p* = 0.020). Similarly, the highest ferritin index tertile was significantly associated with MACE risk (adjusted HR: 1.73, *p* = 0.018), with further strengthening post-IPTW adjustment (HR: 1.92, *p* = 0.003).

**Table 2.** Impact of serum ferritin, ferritin index, and FIB-4 score on MACE event rates, with adjusted and IPTW hazard ratios.

	crude HR (95% CI)	*p* value	adjusted HR (95% CI)	*p* value	IPTW HR (95% CI)	*p* value
serum Ferritin						
Ferritin < 94 (*N* = 245)	1.07(0.66,1.74)	0.775	1.07(0.66,1.74)	0.771	1.06(0.66,1.71)	0.8109
Ferritin in 94 to <315 (*N* = 258)	1 (reference)		1 (reference)		1 (reference)	
Ferritin ≥315 (*N* = 248)	1.63(1.04,2.53)	0.031	1.61(1.03,2.53)	0.038	1.67(1.08,2.59)	0.020
Ferritin index (FI) RI using Beckman two-side immunoassay analytes						
FI < 0.29 (*N* = 245)	1.14(0.70,1.85)	0.591	1.15(0.71,1.85)	0.579	1.12(0.69,1.82)	0.634
FI in 0.29 to <0.94 (*N* = 256)	1 (reference)		1 (reference)		1 (reference)	
FI ≥ 0.94 (*N* = 250)	1.74(1.11,2.72)	0.015	1.73(1.10,2.72)	0.018	1.92(1.25,2.95)	0.003
FIB-4 score						
FIB-4 score <1.45 (*N* = 149)	1 (reference)		1 (reference)		1 (reference)	
FIB-4 score: 1.45 − 3.25 (*N* = 208)	1.21(0.72,2.04)	0.471	1.38(0.79,2.41)	0.256	1.09(0.67,1.78)	0.718
FIB-4 score >3.25 (*N* = 394)	0.87(0.53,1.43)	0.581	1.12(0.63,2.02)	0.696	0.77(0.48,1.24)	0.281

### FIB-4 score and MACE

Kaplan–Meier survival curves did not show significant differences in MACE rates among the three FIB-4 score tertiles (Figure 3C; Table 2).

### Correlation between FIB-4 score and ferritin index

**Figure 4.**
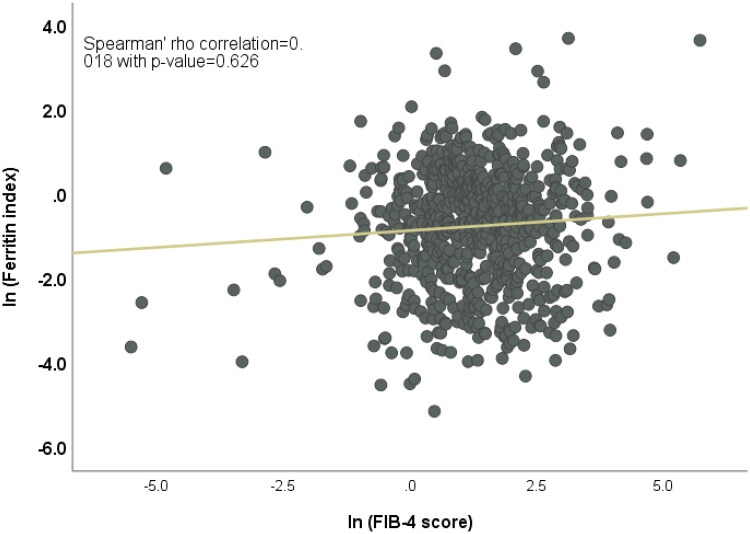
Correlation analysis depicting the relationship between ferritin index (FI) and FIB-4 scores.

No significant correlation was found between FI and the FIB-4 score (Spearman’s rho = 0.18, *p* = 0.26) (Figure 4).

### Evaluation of ferritin, ferritin index, and FIB-4 score as predictors of MACE in heart failure patients

Receiver operating characteristic (ROC) analysis was conducted to explore the discriminatory performance of ferritin, ferritin index, and FIB-4 score for MACE events. While the ferritin index demonstrated statistically significant but modest discrimination (AUC = 0.560, 95% CI: 0.50–0.62, *p* = 0.039), the FIB-4 score had a lower and non-significant AUC (0.471, *p* = 0.320). Given the absence of an external validation cohort and the limited added value of the ROC curves, these findings are available in Supplemental Figure 1.

### Sensitivity and subgroup analyses

Sensitivity analysis (Table 3) excluding patients with pre-existing coronary artery disease (CAD), stroke, or atrial fibrillation (AF) demonstrated that the highest ferritin index tertile remained significantly associated with an increased risk of MACE (adjusted HR: 2.38, *p* = 0.023; IPTW HR: 2.18, *p* = 0.039). A similar trend was observed for serum ferritin levels (adjusted HR: 2.11, *p* = 0.048), although the association attenuated after IPTW adjustment (*p* = 0.063).

**Table 3.** Sensitivity analysis assessing the impact of serum ferritin and ferritin index on MACE event rates among patients without stroke, coronary artery disease, or atrial fibrillation history.

	Adjusted HR (95% CI)	*p* value	IPTW HR (95% CI)	*p* value
serum Ferritin				
Ferritin < 94 (*N* = 245)	1.46(0.65,3.28)	0.358	1.15(0.49,2.68)	0.749
Ferritin in 94 to <315(*N* = 258)	1 (reference)		1 (reference)	
Ferritin ≥315 (*N* = 248)	2.11(1.01,4.41)	0.048	2.03(0.96,4.3)	0.063
Ferritin index (FI) RI using Beckman two-side immunoassay analytes				
FI < 0.29 (*N* = 245)	1.28(0.54,3)	0.576	1.13(0.49,2.65)	0.772
FI in 0.29 to <0.94 (*N* = 256)	1 (reference)		1 (reference)	
FI ≥ 0.94 (*N* = 250)	2.38(1.13,5.02)	0.023	2.18(1.04,4.58)	0.039
FIB-4 score				
FIB-4 score <1.45(*N* = 149)	1 (reference)		1 (reference)	
FIB-4 score: 1.45 − 3.25(*N* = 208)	1.56(0.66,3.68)	0.308	0.88(0.39,1.96)	0.753
FIB-4 score >3.25(*N* = 394)	1.40(0.58,3.35)	0.451	0.78(0.36,1.68)	0.517

**Figure 5.**
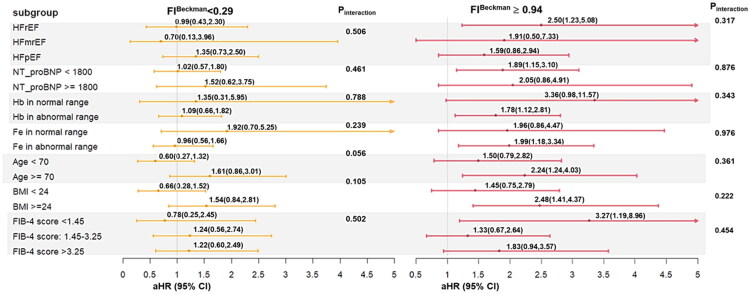
Forest plot illustrating the subgroup analysis of MACE risk across different patient characteristics.

Subgroup analysis (Table 4; Figure 5) evaluated the association between ferritin index tertiles and MACE risk across several strata, using the middle tertile (0.29–0.94) as the reference. Patients aged ≥70 years had a significantly higher MACE risk (adjusted HR: 2.24; 95% CI: 1.24–4.03), as did those with BMI ≥24 (adjusted HR: 2.48; 95% CI: 1.41–4.37). Heart failure with reduced ejection fraction (HFrEF) was associated with an elevated MACE risk (adjusted HR: 2.50; 95% CI: 1.23–5.08). Elevated risk was also observed in patients with NT-proBNP <1800 pg/mL (adjusted HR: 1.89; 95% CI: 1.15–3.10), abnormal hemoglobin (adjusted HR: 1.78; 95% CI: 1.12–2.81), and abnormal serum iron levels (adjusted HR: 1.99; 95% CI: 1.18–3.34). Notably, a FIB-4 score <1.45 was associated with the highest relative risk (adjusted HR: 3.27; 95% CI: 1.19–8.96).

**Table 4.** Subgroup analysis of MACE risk stratified by ferritin index tertiles, evaluating associations across various clinical and demographic characteristics.

Subgroup		FI^Beckman^<0.29	FI^Beckman^ in 0.29 to <0.94	FI^Beckman^ ≥ 0.94
EF type	HFrEF (*N* = 256)	0.99(0.43,2.3)	1 (reference)	2.50(1.23,5.08)
	HFmrEF (*N* = 102)	0.70(0.13,3.96)	1 (reference)	1.91(0.50,7.33)
	HFpEF (*N* = 393)	1.35(0.73,2.50)	1 (reference)	1.59(0.86,2.94)
	*P*_interaction_	0.506		0.317
NT_proBNP (pg/mL)	NT_proBNP < 1800 (*N* = 620)	1.02(0.57,1.80)	1 (reference)	1.89(1.15,3.10)
	NT_proBNP ≥ 1800 (*N* = 131)	1.52(0.62,3.75)	1 (reference)	2.05(0.86,4.91)
	*P*_interaction_	0.461		0.876
Hb normal range in Female: 11-16(g/dL); Male: 13-18(g/dL)	Hb in normal range (*N* = 123)	1.35(0.31,5.95)	1 (reference)	3.36(0.98,11.57)
	Hb in abnormal range (*N* = 628)	1.09(0.66,1.82)	1 (reference)	1.78(1.12,2.81)
	*P*_interaction_	0.788		0.343
Fe normal range in 50-212(μg/dL)	Fe in normal range (*N* = 223)	1.92(0.70,5.25)	1 (reference)	1.96(0.86,4.47)
	Fe in abnormal range (*N* = 528)	0.96(0.56,1.66)	1 (reference)	1.99(1.18,3.34)
	*P*_interaction_	0.239		0.976
Age category	Age < 70 (*N* = 276)	0.6(0.27,1.32)	1 (reference)	1.50(0.79,2.82)
	Age ≥ 70 (*N* = 475)	1.61(0.86,3.01)	1 (reference)	2.24(1.24,4.03)
	*P*_interaction_	0.056		0.361
BMI category(kg/m^2^)	BMI < 24 (*N* = 375)	0.66(0.28,1.52)	1 (reference)	1.45(0.75,2.79)
	BMI ≥ 24 (*N* = 376)	1.54(0.84,2.81)	1 (reference)	2.48(1.41,4.37)
	*P*_interaction_	0.1051		0.2218
FIB-4 score	FIB-4 score <1.45 (*N* = 149)	0.78(0.25,2.45)	1 (reference)	3.27(1.19,8.96)
	FIB-4 score: 1.45 − 3.25 (*N* = 208)	1.24(0.56,2.74)	1 (reference)	1.33(0.67,2.64)
	FIB-4 score >3.25 (*N* = 394)	1.22(0.60,2.49)	1 (reference)	1.83(0.94,3.57)
	*P*_interaction_	0.5018		0.4542

Additional sensitivity analyses for transferrin saturation (TSAT), C-reactive protein (CRP), and NT-proBNP are presented in Supplementary Tables 3, 4 and Supplementary Figure 2. These analyses may have been underpowered due to missing data, particularly for TSAT and CRP. For transparency, we also provide sensitivity analyses excluding new-onset atrial fibrillation from the MACE definition in the Supplementary Table 5.

## Discussion

### Ferritin index as a risk stratifier in patients with heart failure

In this cohort study with a median follow-up duration of 1.48 years (Interquartile range: 0.57–3.44 years), we investigated the prognostic value of baseline serum ferritin levels and the ferritin index in predicting major adverse cardiac events (MACE) among heart failure patients with varying left ventricular ejection fractions. Our findings demonstrate that elevated serum ferritin levels and a higher ferritin index are associated with increased MACE incidence. Notably, the ferritin index demonstrated superior predictive performance compared to serum ferritin alone (log-rank *p* = 0.02 vs. *p* = 0.046), supporting its potential utility as a biomarker for risk stratification in heart failure.

Sensitivity analyses reinforced the robustness of these associations even after excluding patients with pre-existing coronary artery disease, stroke, or atrial fibrillation. The ferritin index effectively identified at-risk individuals, including those with low FIB-4 scores (<1.45) (aHR = 3.27, 95% CI = 1.19–8.96) and relatively lower NT-proBNP levels (<1800) (aHR = 1.89, 95% CI = 1.15–3.10) (Table 4). Given that serum ferritin reference values vary by age, sex, and laboratory methods, we calculated the ferritin index (FI = observed serum ferritin/upper limit of normal ferritin concentration for age and sex) to establish physiologically relevant thresholds that support clinical decision-making [28]. While the ferritin index has been previously investigated in septic shock prognosis [25], this study is among the first to explore its prognostic relevance in heart failure.

Given the lack of established cutoff values for ferritin index-based risk stratification in this population, we adopted a tertile-based approach to facilitate exploratory analysis. However, we acknowledge the need for future studies to define biologically and clinically meaningful thresholds to optimize its clinical applicability.

Our findings are consistent with prior studies, including the EDIFICA cohort [11], which identified elevated ferritin as an independent predictor of poor outcomes in acute heart failure, regardless of key iron, inflammatory, and prognostic markers.

Recent evidence by De Biase et al. [29] underscores the limitations of ferritin as an isolated biomarker, due to its modulation by systemic inflammation and poor reflection of true iron status. In their study, transferrin saturation (TSAT) <20% and serum iron ≤13 μmol/L were more strongly linked to congestion, reduced exercise capacity, and adverse outcomes than traditional ferritin-based definitions of iron deficiency. These findings highlight the clinical relevance of using integrated markers like the ferritin index, which normalizes ferritin to age- and sex-specific reference limits and potentially provides a more physiologically meaningful measure that captures both iron homeostasis and inflammation. This supports our assertion that the ferritin index may offer superior risk stratification in heart failure patients by bridging inflammation, metabolic dysfunction, and hepatic involvement.

### Comparison of the ferritin index and FIB-4 score in predicting MACE

Heart failure is a multisystemic disease, with the liver being particularly susceptible to congestion. The bidirectional relationship between heart failure and liver dysfunction is well-documented, with a prevalence reaching 65% [30]. Liver congestion may contribute to fibrosis and adverse outcomes [31]. The FIB-4 index, commonly used to assess liver fibrosis and stiffness, has been shown to predict all-cause mortality and cardiovascular events in heart failure [8,9,32]. However, in our study, the FIB-4 score did not effectively stratify MACE risk across its tertiles (Figure 3C), whereas the ferritin index demonstrated superior predictive performance.

Several factors may explain this disparity. Unlike the FIB-4 index, which serves as a liver-specific biomarker, the ferritin index integrates inflammatory, metabolic, and hepatic biomarkers.

Importantly, our findings align with the emerging recognition of metabolic hyperferritinaemia (MHF) as a clinically meaningful phenotype [15]. MHF refers to elevated serum ferritin levels occurring in the absence of iron overload but in the presence of metabolic dysfunction such as obesity, type 2 diabetes, or dyslipidemia. This phenotype reflects a pro-inflammatory state characterized by iron sequestration within macrophages, preserved hepcidin levels, and dysregulated iron homeostasis—pathways increasingly implicated in the progression of both cardiovascular and liver disease. The ferritin index may thus serve as a surrogate for MHF, providing insight into a metabolically driven, inflammatory milieu that may not be adequately captured by liver-specific indices such as FIB-4.

Moreover, the frequent co-occurrence of metabolic dysfunction-associated steatotic liver disease (MASLD) in heart failure (HF) populations further supports the clinical relevance of metabolic hyperferritinaemia (MHF) [33,34]. MASLD is now considered a central feature of cardio-metabolic multimorbidity [35], and MHF is likely to represent a biomarker of disease severity within this context [13,14]. The ferritin index may therefore function not only as a prognostic tool but also as a pathophysiological bridge linking inflammation, liver dysfunction, and metabolic disease in HF.

Although FIB-4 and the ferritin index were developed for different clinical applications—liver fibrosis assessment and systemic inflammation/metabolism, respectively—both are readily available in clinical practice and increasingly evaluated in HF populations. Direct comparison of their prognostic utility in HF patients helps clarify which biomarker better captures the multisystem disease burden and offers more actionable risk stratification for clinicians.

These insights may have therapeutic implications. Targeting metabolic inflammation and iron dysregulation—rather than focusing solely on hepatic fibrosis—could represent a more effective strategy in this high-risk population. Further studies are needed to evaluate whether interventions that modulate iron metabolism or systemic inflammation can alter outcomes in patients with elevated ferritin index and MHF features.

### Ferritin index as a pathophysiological bridge

A growing body of evidence suggests that hepatic dysfunction, systemic inflammation, and iron sequestration act along a unified pathophysiological axis in heart failure. Inflammatory signaling—particularly *via* interleukin-6—stimulates hepcidin expression, which promotes iron retention within macrophages, impairs hepatic iron export, and contributes to myocardial remodeling, fibrosis, and metabolic stress [18–23]. This multi-organ interplay underscores the biological plausibility of using integrative biomarkers such as the ferritin index to capture risk and disease severity in HF beyond traditional cardiac parameters.

Despite its clinical relevance, the heart-liver axis remains underappreciated in routine HF management. A more integrated understanding of hepatic involvement in HF has the potential to enhance clinical decision-making and improve outcomes across the spectrum of cardiometabolic disease. The ferritin index—unlike liver-specific tools like FIB-4—offers a multidimensional view, incorporating inflammation, metabolic dysfunction, and hepatic signals.

### New onset AF in MACE

In defining MACE, we included new-onset AF in addition to hospitalization for heart failure, acute myocardial infarction, and stroke. Although this inclusion is unconventional, it reflects the evolving clinical significance of new-onset AF in HF. New-onset AF in HF patients is not merely an arrhythmic event; it is associated with significantly higher risks of heart failure hospitalization, cardiovascular death, renal decline, and all-cause mortality [36–38]. Recent high-impact trials, including the FINE-HEART pooled analysis [39], have prospectively adjudicated new-onset AF as a key secondary endpoint across the cardio-kidney-metabolic (CKM) spectrum. In this analysis of over 14,000 patients, new-onset AF/AFL was independently associated with a 3.7-fold higher risk of HF hospitalization or CV death, and nearly 2.8-fold higher risk of MACE.

These findings underscore its emerging importance as both a therapeutic target and prognostic marker. Moreover, guidelines and consensus documents increasingly recognize new-onset atrial fibrillation as a manifestation of worsening systemic and cardiorenal-metabolic dysfunction, especially in HFpEF patients [40,41]. Its inclusion in our analysis allows for a more comprehensive assessment of adverse cardiovascular trajectories in this complex patient population and reflects real-world clinical progression.

To address concerns regarding endpoint definition, we conducted a sensitivity analysis excluding new-onset AF from the MACE definition, as presented in Supplemental Table 5. The results remained consistent, supporting the validity of our primary findings regardless of endpoint specification.

### Limitations

Although the ferritin index demonstrated a statistically significant association with MACE in both adjusted and IPTW analyses, its discriminatory capacity, as reflected by the AUC of 0.560 (Supplemental Figure 1), remains modest. We acknowledge that this level of performance is insufficient for clinical implementation and should be interpreted cautiously. The ROC findings primarily serve an exploratory purpose and underscore the need for future studies involving multi-marker approaches and external validation cohorts. The ferritin index, at present, is best viewed as a hypothesis-generating marker that captures the interplay of systemic inflammation, metabolic dysregulation, and hepatic dysfunction in heart failure, rather than a definitive predictive model. Future prospective studies with external validation are warranted to assess the generalizability and clinical utility of this index.

Another major limitation of our study is the exclusion of patients without available ferritin testing, which accounted for 77% of the initial cohort. While this raises the potential for selection bias, it is reflective of real-world clinical practice, where iron studies are inconsistently ordered despite guideline recommendations. In line with this, a recent Canadian population-based study by Wahid et al. [42] reported similarly low testing rates—only 38.5% in acute HF and 34.2% in chronic HF cohorts—highlighting a pervasive underuse of iron screening in HF care. Thus, while our findings may have limited generalizability, they provide meaningful insight into a population subset where iron metabolism was clinically assessed.

### Other limitations

1. The retrospective design may introduce selection bias and limits causal inference.
2. The study was conducted at a single medical center in Taiwan, potentially limiting generalizability to other populations.
3. The median follow-up duration of 1.48 years may not fully capture the long-term implications of ferritin levels on MACE.
4. While the ferritin index showed promise, its predictive utility in combination with other biomarkers remains unexplored.
5. The study population primarily consisted of older adults, limiting applicability to younger patients.
6. The timing of ferritin measurement (inpatient vs. outpatient) was not standardized, which may introduce variability in biomarker interpretation. However, this reflects real-world clinical practice, and we accounted for potential confounding through IPW and multivariable adjustment to preserve internal validity.
7. The study did not incorporate a mechanistic evaluation of the interactions between iron metabolism, inflammation, and liver function. Future prospective studies with molecular and histopathologic assessments are warranted to clarify these complex relationships.

### Strengths

1. The study utilized a large clinical database from Changhua Christian Hospital, ensuring real-world applicability.
2. The ferritin index, a novel biomarker combining inflammatory and liver-specific markers, was evaluated as a predictor of MACE.
3. A substantial cohort of 751 heart failure patients provided adequate statistical power.
4. The study design allowed for head-to-head comparison of the ferritin index and the FIB-4 score, highlighting the superiority of a multi-biomarker approach over a liver-specific biomarker alone, offering clinically actionable insights

## Conclusion

The ferritin index outperforms the FIB-4 score in predicting MACE in HF patients and likely reflects broader pathophysiological processes beyond hepatic fibrosis. By integrating features of metabolic hyperferritinaemia (MHF), the ferritin index may serve as a practical, readily available biomarker for identifying high-risk HF patients with underlying metabolic-inflammatory dysregulation. This approach aligns with the evolving recognition of HF as a multisystem syndrome and highlights the importance of interdisciplinary risk assessment strategies.

## Supplementary Material

Supplemental Material

Supplementary Figure 1.docx

Supplementary Table 3.docx

Supplementary Table 4.docx

Supplementary Table 1.docx

Supplementary Table 5.docx

Supplementary Table 2.docx

## Data Availability

The derived data that were generated in the current study are available from the corresponding author upon reasonable request.
