# Supplementary material for: Integrating systemic inflammation and liver biomarkers: prognostic implications of the ferritin index in heart failure
Source: Ann Med. 2025 Aug 1;57(1):2540020. doi: 10.1080/07853890.2025.2540020 (PMC12320259; doi:10.1080/07853890.2025.2540020)

**Supplemental Figure 2**. Kaplan-Meier curves illustrating MACE incidence rates among CRP and NT-proBNP tertiles.


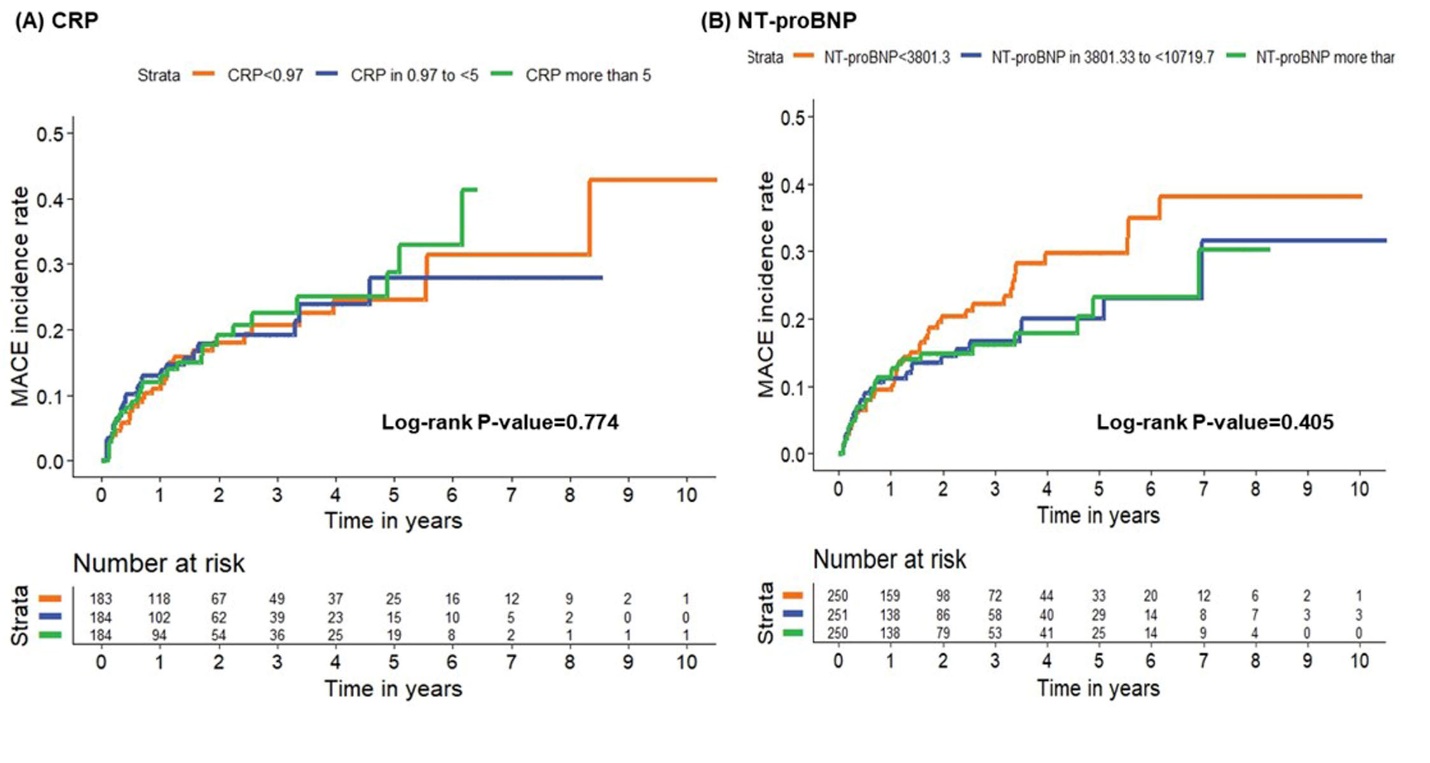

Supplement: Supplemental Material [file IANN_A_2540020_SM3899.docx]
