# Supplementary material for: Integrating systemic inflammation and liver biomarkers: prognostic implications of the ferritin index in heart failure
Source: Ann Med. 2025 Aug 1;57(1):2540020. doi: 10.1080/07853890.2025.2540020 (PMC12320259; doi:10.1080/07853890.2025.2540020)

**Supplemental Figure 1**. **Receiver operating characteristic (ROC) curves evaluating the predictive performance of serum ferritin, ferritin index, and FIB-4 score for MACE events**


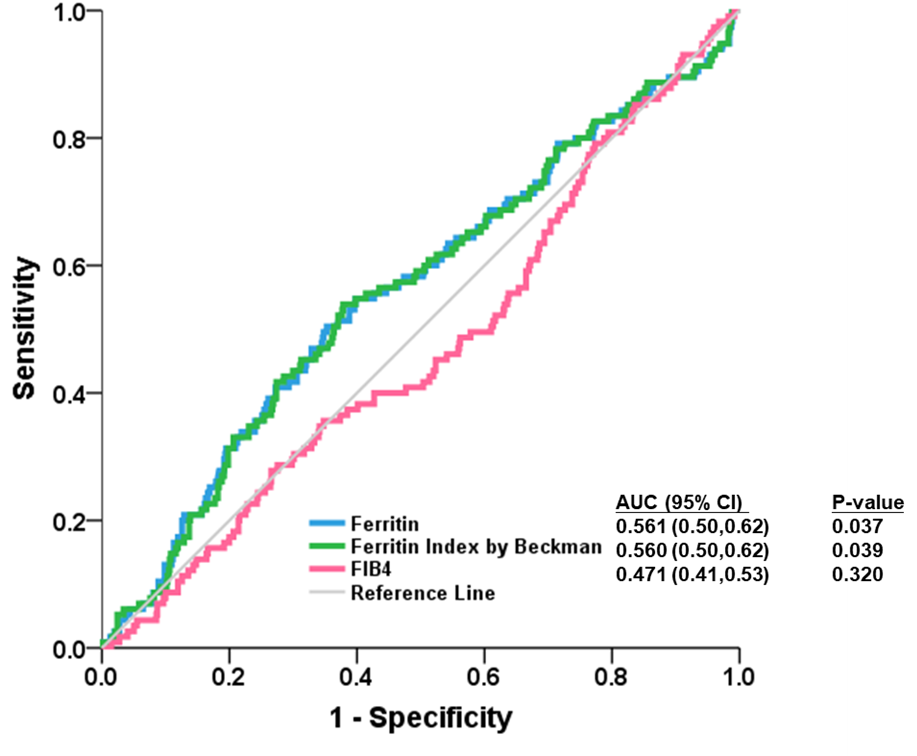

Supplement: Supplementary Figure 1.docx [file IANN_A_2540020_SM4450.docx]
