# Supplementary material for: Integrating systemic inflammation and liver biomarkers: prognostic implications of the ferritin index in heart failure
Source: Ann Med. 2025 Aug 1;57(1):2540020. doi: 10.1080/07853890.2025.2540020 (PMC12320259; doi:10.1080/07853890.2025.2540020)
Supplement: Supplementary Table 3.docx [file IANN_A_2540020_SM4449.docx]

**Supplemental Table 3. Subgroup analysis of MACE risk stratified by ferritin index tertiles, evaluating associations across various clinical and demographic characteristics including transferrin saturation**

| Subgroup | | FI^Beckman^<0.29 | FI^Beckman^ in 0.29 to <0.94 | FI^Beckman^ ≥ 0.94 |
| --- | --- | --- | --- | --- |
| EF type | HFrEF (N=256) | 0.99(0.43,2.3) | 1 (reference) | 2.50(1.23,5.08) |
|  | HFmrEF (N=102) | 0.70(0.13,3.96) | 1 (reference) | 1.91(0.50,7.33) |
|  | HFpEF (N=393) | 1.35(0.73,2.50) | 1 (reference) | 1.59(0.86,2.94) |
|  | Pinteraction | 0.506 |  | 0.317 |
| NT_proBNP (pg/mL) | NT_proBNP < 1800 (N=620) | 1.02(0.57,1.80) | 1 (reference) | 1.89(1.15,3.10) |
|  | NT_proBNP ≥ 1800 (N=131) | 1.52(0.62,3.75) | 1 (reference) | 2.05(0.86,4.91) |
|  | Pinteraction | 0.461 |  | 0.876 |
| Hb normal range in Female: 11-16(g/dL);  Male: 13-18(g/dL) | Hb in normal range (N=123) | 1.35(0.31,5.95) | 1 (reference) | 3.36(0.98,11.57) |
|  | Hb in abnormal range (N=628) | 1.09(0.66,1.82) | 1 (reference) | 1.78(1.12,2.81) |
|  | Pinteraction | 0.788 |  | 0.343 |
| Fe normal range in 50-212(μg/dL) | Fe in normal range (N=223) | 1.92(0.70,5.25) | 1 (reference) | 1.96(0.86,4.47) |
|  | Fe in abnormal range (N=528) | 0.96(0.56,1.66) | 1 (reference) | 1.99(1.18,3.34) |
|  | Pinteraction | 0.239 |  | 0.976 |
| Age category | Age < 70 (N=276) | 0.6(0.27,1.32) | 1 (reference) | 1.50(0.79,2.82) |
|  | Age ≥ 70 (N=475) | 1.61(0.86,3.01) | 1 (reference) | 2.24(1.24,4.03) |
|  | Pinteraction | 0.056 |  | 0.361 |
| BMI category(kg/m^2^) | BMI < 24 (N=375) | 0.66(0.28,1.52) | 1 (reference) | 1.45(0.75,2.79) |
|  | BMI ≥ 24 (N=376) | 1.54(0.84,2.81) | 1 (reference) | 2.48(1.41,4.37) |
|  | Pinteraction | 0.1051 |  | 0.2218 |
| FIB-4 score | FIB-4 score <1.45 (N=149) | 0.78(0.25,2.45) | 1 (reference) | 3.27(1.19,8.96) |
|  | FIB-4 score: 1.45−3.25 (N=208) | 1.24(0.56,2.74) | 1 (reference) | 1.33(0.67,2.64) |
|  | FIB-4 score >3.25 (N=394) | 1.22(0.60,2.49) | 1 (reference) | 1.83(0.94,3.57) |
|  | Pinteraction | 0.5018 |  | 0.4542 |
| TSAT | TSAT < 20 (N=150) | 1.82 (0.69,4.76) | 1 (reference) | 3.81(1.47,9.85) |
|  | TSAT ≥ 20 (N=149) | 0.60(0.12,3.13) | 1 (reference) | 0.92(0.43,2.00) |
|  | Pinteraction | 0.2563 |  | 0.0231 |
