# Supplementary material for: Integrating systemic inflammation and liver biomarkers: prognostic implications of the ferritin index in heart failure
Source: Ann Med. 2025 Aug 1;57(1):2540020. doi: 10.1080/07853890.2025.2540020 (PMC12320259; doi:10.1080/07853890.2025.2540020)
Supplement: Supplementary Table 4.docx [file IANN_A_2540020_SM4448.docx]

**Supplementary Table 4. Sensitivity analysis assessing the impact of C-reactive protein (CRP), and NT-pro-BNP on MACE event rates**

|  | crude HR (95% CI) | P-value | adjusted HR (95% CI) | P-value | IPTW HR (95% CI) | P-value |
| --- | --- | --- | --- | --- | --- | --- |
| serum Ferritin |  |  |  |  |  |  |
| Ferritin<94 (N=245) | 1.07(0.66,1.74) | 0.775 | 1.07(0.66,1.74) | 0.771 | 1.06(0.66,1.71) | 0.8109 |
| Ferritin in 94 to <315 (N=258) | 1 (reference) |  | 1 (reference) |  | 1 (reference) |  |
| Ferritin ≥315 (N=248) | 1.63(1.04,2.53) | 0.031 | 1.61(1.03,2.53) | 0.038 | 1.67(1.08,2.59) | 0.020 |
| Ferritin index (FI) RI using Beckman two-side immunoassay analytes | |  |  |  |  |  |
| FI<0.29 (N=245) | 1.14(0.70,1.85) | 0.591 | 1.15(0.71,1.85) | 0.579 | 1.12(0.69,1.82) | 0.634 |
| FI in 0.29 to <0.94 (N=256) | 1 (reference) |  | 1 (reference) |  | 1 (reference) |  |
| FI ≥ 0.94 (N=250) | 1.74(1.11,2.72) | 0.015 | 1.73(1.10,2.72) | 0.018 | 1.92(1.25,2.95) | 0.003 |
| FIB-4 score |  |  |  |  |  |  |
| FIB-4 score <1.45 (N=149) | 1 (reference) |  | 1 (reference) |  | 1 (reference) |  |
| FIB-4 score: 1.45−3.25 (N=208) | 1.21(0.72,2.04) | 0.471 | 1.38(0.79,2.41) | 0.256 | 1.09(0.67,1.78) | 0.718 |
| FIB-4 score >3.25 (N=394) | 0.87(0.53,1.43) | 0.581 | 1.12(0.63,2.02) | 0.696 | 0.77(0.48,1.24) | 0.281 |
| NT-proBNP |  |  |  |  |  |  |
| NT-proBNP<3801.3 (N=250) | 1 (reference) |  | 1 (reference) |  | 1 (reference) |  |
| NT-proBNP: 3801.3 to <10719.67 (N=251) | 0.69(0.45,1.07) | 0.101 | 0.71(0.46,1.1) | 0.124 | 0.66(0.41,1.07) | 0.091 |
| NT-proBNP > 10719.67 (N=250) | 0.73(0.47,1.12) | 0.147 | 0.69(0.45,1.07) | 0.099 | 0.68(0.41,1.1) | 0.118 |
| CRP |  |  |  |  |  |  |
| CRP<0.97 (N=183) | 1 (reference) |  | 1 (reference) |  | 1 (reference) |  |
| CRP: 0.97 to <5 (N=184) | 0.96(0.58,1.57) | 0.859 | 1(0.61,1.66) | 0.988 | 0.96(0.58,1.6) | 0.888 |
| CRP ≥ 5 (N=184) | 1.07(0.66,1.73) | 0.772 | 1.19(0.74,1.91) | 0.483 | 1.09(0.66,1.8) | 0.728 |
