# Supplementary material for: Integrating systemic inflammation and liver biomarkers: prognostic implications of the ferritin index in heart failure
Source: Ann Med. 2025 Aug 1;57(1):2540020. doi: 10.1080/07853890.2025.2540020 (PMC12320259; doi:10.1080/07853890.2025.2540020)
Supplement: Supplementary Table 1.docx [file IANN_A_2540020_SM4447.docx]

**Supplementary Table 1**. **Comparison of demographic and clinical characteristics between the inclusion cohort (n = 751) and exclusion cohort (n = 2,542)**

| Sample size | 2542 | 751 | P-value |
| --- | --- | --- | --- |
| LVEF (%) | 43.6±18.3 | 49.5±17 | <0.001 |
| Age(years) | 72±14.8 | 72.5±14.4 | 0.378 |
| Gender, male | 1400(55.1%) | 361(48.1%) | 0.001 |
| BMI (kg/m^2^) | 25.8±8.4 | 26±12.6 | 0.696 |
| NT pro-BNP (pg/mL) | 6222.5±6755.4 | 9377.8±8600 | <0.001 |
| Comorbidity disease |  |  |  |
| DM | 1053(41.4%) | 435(57.9%) | <0.001 |
| Hypertension | 1256(49.4%) | 502(66.8%) | <0.001 |
| Hyperlipidemia | 686(27%) | 294(39.1%) | <0.001 |
| Coronary artery disease | 671(26.4%) | 246(32.8%) | 0.001 |
| COPD | 364(14.3%) | 127(16.9%) | 0.080 |
| CKD | 379(14.9%) | 346(46.1%) | <0.001 |
| Atrial fibrillation | 608(23.9%) | 197(26.2%) | 0.195 |
| Stroke | 423(16.6%) | 159(21.2%) | 0.004 |
| Medication use in hypertension |  |  |  |
| ACEARB | 2107(82.9%) | 628(83.6%) | 0.637 |
| Alpha blocker | 161(6.3%) | 92(12.3%) | <0.001 |
| Beta blocker | 1795(70.6%) | 556(74%) | 0.068 |
| Calcium channel blocker | 1313(51.7%) | 485(64.6%) | <0.001 |
| Thiazide | 290(11.4%) | 102(13.6%) | 0.106 |
| Loop diuretics | 2203(86.7%) | 661(88%) | 0.334 |
| Spironolactone | 1388(54.6%) | 324(43.1%) | <0.001 |
| Anti-DM medication |  |  |  |
| Statin | 1010(39.7%) | 296(39.4%) | 0.875 |
| Aspirin | 1654(65.1%) | 588(78.3%) | <0.001 |
| NSAID | 64(2.5%) | 26(3.5%) | 0.163 |
| Lab data |  |  |  |
| HbA1c (%) | 6.8±1.3 | 6.7±1.4 | 0.003 |
| Hb (g/dL) | 12.2±2.3 | 9.6±2.7 | <0.001 |
| Creatinine (mg/ dL) | 1.8±4.7 | 3.5±3.4 | <0.001 |
| Albumin (g/dL) | 3.2±0.5 | 3.1±0.7 | <0.001 |
| WBC count (10^3^ /μL) | 9.3±4.2 | 9.4±5.7 | 0.677 |
| K (mmol/L) | 4.1±2.8 | 4.5±5.1 | 0.039 |
| Estimated GFR (mL / min / 1.73m^2^) | 57.5±30.9 | 38.5±35.8 | <0.001 |
| Na (mmol/L) | 135.9±6.1 | 134.6±8.6 | <0.001 |
| Platelet count (10^3^ /μL) | 207.1±85.9 | 211.7±103.6 | 0.269 |
| RDW (%) | 4.1±0.9 | 3.4±1 | <0.001 |
| GPT(ALT) (U/L) | 53.4±205.3 | 37.7±142.7 | 0.018 |
| GOT(AST) (U/L) | 78.7±346.5 | 54.9±206.7 | 0.020 |
| Cholesterol (mg/dL) | 158.6±33.4 | 155±41.7 | 0.029 |
| Triglyceride (mg/dL) | 122.7±68.6 | 124.6±79.9 | 0.522 |
| LDL cholesterol (mg/dL) | 92.2±26.8 | 87.9±32 | 0.001 |
| HDL cholesterol (mg/dL) | 41.4±10 | 39.7±10.6 | <0.001 |
| APTT (sec.) | 33.4±9.8 | 33±9.6 | 0.335 |
