# Supplementary material for: Integrating systemic inflammation and liver biomarkers: prognostic implications of the ferritin index in heart failure
Source: Ann Med. 2025 Aug 1;57(1):2540020. doi: 10.1080/07853890.2025.2540020 (PMC12320259; doi:10.1080/07853890.2025.2540020)
Supplement: Supplementary Table 5.docx [file IANN_A_2540020_SM4446.docx]

**Supplementary Table 5. Sensitivity Analysis of MACE Excluding New-Onset Atrial Fibrillation**

|  | crude HR (95% CI) | P-value | adjusted HR (95% CI) | P-value | IPTW HR (95% CI) | P-value |
| --- | --- | --- | --- | --- | --- | --- |
| serum Ferritin |  |  |  |  |  |  |
| Ferritin<94 (N=245) | 1.08(0.73,1.59) | 0.700 | 1.11(0.76,1.64) | 0.584 | 0.94(0.61,1.44) | 0.760 |
| Ferritin in 94 to <315 (N=258) | 1 (reference) |  |  |  |  |  |
| Ferritin ≥315 (N=248) | 1.48(1.03,2.13) | 0.036 | 1.65(1.14,2.4) | 0.008 | 1.5(1,2.23) | 0.049 |
| Ferritin index (FI) RI using Beckman two-side immunoassay analytes | | |  |  |  |  |
| FI<0.29 (N=245) | 1.17(0.79,1.72) | 0.444 | 1.18(0.8,1.75) | 0.404 | 0.99(0.64,1.54) | 0.979 |
| FI in 0.29 to <0.94 (N=256) |  |  |  |  |  |  |
| FI ≥ 0.94 (N=250) | 1.63(1.13,2.35) | 0.010 | 1.77(1.22,2.58) | 0.003 | 1.64(1.1,2.45) | 0.016 |
| FIB-4 score |  |  |  |  |  |  |
| FIB-4 score <1.45 (N=149) | 1 (reference) |  | 1 (reference) |  | 1 (reference) |  |
| FIB-4 score: 1.45−3.25 (N=208) | 1.54(0.96,2.46) | 0.071 | 1.4(0.87,2.26) | 0.164 | 1.12(0.72,1.74) | 0.626 |
| FIB-4 score >3.25 (N=394) | 1.31(0.84,2.05) | 0.235 | 1.16(0.73,1.83) | 0.540 | 1.3(0.86,1.98) | 0.219 |
