# Supplementary material for: Integrating systemic inflammation and liver biomarkers: prognostic implications of the ferritin index in heart failure
Source: Ann Med. 2025 Aug 1;57(1):2540020. doi: 10.1080/07853890.2025.2540020 (PMC12320259; doi:10.1080/07853890.2025.2540020)
Supplement: Supplementary Table 2.docx [file IANN_A_2540020_SM4444.docx]

**Supplemental Table 2.** **Impact of serum ferritin, ferritin index, and FIB-4 score on MACE event rates, with adjusted and IPTW hazard ratios.** **The analysis was truncated at 6 years to reflect the follow-up distribution (median: 1.48 years; IQR: 0.57–3.44 years).**

| 6-year follow up | crude HR (95% CI) | P-value | adjusted HR (95% CI) | P-value | IPTW HR (95% CI) | P-value |
| --- | --- | --- | --- | --- | --- | --- |
| serum Ferritin |  |  |  |  |  |  |
| Ferritin<94 (N=245) | 1.08(0.67,1.74) | 0.765 | 1.08(0.67,1.76) | 0.744 | 1.02(0.63,1.66) | 0.930 |
| Ferritin in 94 to <315 (N=258) | 1 (reference) |  | 1 (reference) |  | 1 (reference) |  |
| Ferritin ≥315 (N=248) | 1.51(0.96,2.38) | 0.073 | 1.51(0.95,2.42) | 0.084 | 1.61(1.03,2.51) | 0.038 |
| Ferritin index (FI) RI using Beckman two-side immunoassay analytes | | |  |  |  |  |
| FI<0.29 (N=245) | 1.18(0.72,1.92) | 0.513 | 1.17(0.72,1.92) | 0.528 | 1.09(0.67,1.76) | 0.730 |
| FI in 0.29 to <0.94 (N=256) | 1 (reference) |  | 1 (reference) |  |  |  |
| FI ≥ 0.94 (N=250) | 1.7(1.08,2.68) | 0.023 | 1.69(1.05,2.73) | 0.030 | 1.69(1.08,2.64) | 0.021 |
| FIB-4 score |  |  |  |  |  |  |
| FIB-4 score <1.45 (N=149) | 1 (reference) |  | 1 (reference) |  | 1 (reference) |  |
| FIB-4 score: 1.45−3.25 (N=208) | 1.25(0.74,2.13) | 0.403 | 1.22(0.72,2.07) | 0.463 | 1.08(0.59,1.97) | 0.800 |
| FIB-4 score >3.25 (N=394) | 0.87(0.52,1.46) | 0.607 | 0.96(0.57,1.61) | 0.883 | 0.87(0.44,1.71) | 0.683 |
